# The Preoperative Assessment of Hepatic Tumours: Evaluation of UK Regional Multidisciplinary Team Performance

**DOI:** 10.1155/2013/861681

**Published:** 2013-08-22

**Authors:** M. G. Wiggans, S. A. Jackson, B. M. T. Fox, J. D. Mitchell, S. Aroori, M. J. Bowles, E. M. Armstrong, J. F. Shirley, D. A. Stell

**Affiliations:** ^1^Plymouth Hospitals NHS Trust, Derriford Hospital, Derriford Road, Plymouth, Devon PL6 8DH, UK; ^2^Plymouth University, Peninsula College of Medicine and Dentistry, John Bull Building, Plymouth, Devon PL6 8BU, UK

## Abstract

*Introduction*. In the UK, patients where liver resection is contemplated are discussed at hepatobiliary multidisciplinary team (MDT) meetings. The aim was to assess MDT performance by identification of patients where radiological and pathological diagnoses differed. *Materials and Methods*. A retrospective review of a prospectively maintained database of all cases undergoing liver resection from March 2006 to January 2012 was performed. The presumed diagnosis as a result of radiological investigation and MDT discussion is recorded at the time of surgery. Imaging was reviewed by specialist gastrointestinal radiologists, and resultswereagreedonby consensus. *Results*. Four hundred and thirty-eight patients were studied. There was a significant increase in the use of preoperative imaging modalities (*P* ≤ 0.01) but no change in the rate of discrepant diagnosis over time. Forty-two individuals were identified whose final histological diagnosis was different to that following MDT discussion (9.6%). These included 30% of patients diagnosed preoperatively with hepatocellular carcinoma and 25% with cholangiocarcinoma of a major duct. *Discussion*. MDT assessment of patients preoperatively is accurate in terms of diagnosis. The highest rate of discrepancies occurred in patients with focal lesions without chronic liver disease or primary cancer, where hepatocellular carcinoma was overdiagnosed and peripheral cholangiocarcinoma underdiagnosed, where particular care should be taken. Additional care should be taken in these groups and preoperative multimodality imaging considered.

## 1. Introduction 

Cancer care in the UK has undergone a major change in recent years with the centralisation of care in a network of cancer centres [1]. This has led to the establishment of regional hepatopancreaticobiliary (HPB) units where patients in whom liver resection is contemplated are discussed at a multidisciplinary team (MDT) meeting in the presence of radiologists, oncologists, surgeons, and physicians. This is intended to provide greater clinical input into the diagnosis of the wide spectrum of disease processes for which liver resection is appropriate [2]. During the same period increasing awareness of the complimentary role of different imaging modalities in diagnosing liver disease [3–5] has led to many patients having multiple investigations prior to surgery. Although the accuracy of single imaging modalities including ultrasound [3, 6, 7], computerised tomography (CT) [3, 7, 8], magnetic resonance imaging (MRI) [3, 7, 9], and positron emission tomography (PET) [3, 8] scans in assessing hepatic malignancies has been well described, the performance of MDT review of multiple preoperative imaging techniques with input from clinicians in the diagnosis of malignancy and planning of treatment has not been described.

The Peninsula HPB unit was founded in July 2005 to serve the Devon and Cornwall region of England (population 1.7 million). Imaging from referring hospitals is imported and discussed in a weekly MDT meeting, and treatment recommendations are made and recorded. After resection histology of the excised sample is also discussed at the MDT meeting. Despite MDT assessment, we have experienced cases either where the histological diagnosis has differed from the presumed preoperative diagnosis or where the available imaging does not allow a certain diagnosis to be made. In this situation a list of differential diagnoses is made from which treatment is recommended. Furthermore, despite advanced imaging techniques, some patients undergo surgery without proceeding to resection due to unexpected operative findings. The primary aim of this study was to identify patients where the diagnosis determined by the MDT differed from the final histological diagnosis. A secondary aim was to identify recurring areas of confusion to guide future MDT assessment and to determine if the rate of inaccurate diagnosis of liver tumours and assessments of resectability of liver lesions has changed over time.

## 2. Materials and Methods

The Peninsula HPB unit has maintained a prospective database since the inception of the unit where the outcome of MDT discussion is recorded prior to surgery. A review of all patients undergoing surgery from March 2006 to January 2012 was performed. Details of preoperative diagnosis, imaging modalities performed, operative findings, and final histology were retrieved. Patients were identified where the MDT was unable to make a definitive diagnosis leading to differential options. All imaging was re-reviewed by a specialist gastrointestinal radiologist and results agreed by consensus. For comparison of utilisation of imaging modalities, the group was split into two halves consisting of 219 patients each. The dataset was also divided to compare the earlier with later experience. Statistical analysis was performed using a chisquare test or Mann-Whitney *U* test, and a *P* value of <0.05 was considered statistically significant. Analyses were performed using SPSS version 20 (IBM, New York, USA).

## 3. Results

### 3.1. Patient Population

Four hundred and thirty-eight patients were identified including 248 males and 190 females with median age 65 years (range 21–90). The indications for surgery are shown in Table 1. Four hundred and seventeen patients underwent liver resection (95%), and 21 patients (5%) underwent surgery without resection. Details of the group not proceeding to resection are shown in Table 2.

### 3.2. Imaging Performed

In total 969 imaging investigations (excluding repeat images of the same modality) were performed for the 438 patients including CT, MRI, PET, US, and ERCP. Only five patients did not have a CT scan. The number of MRI scans undertaken increased from 96 in the first half of the study (219 patients) to 131 in the second the second (*P* = 0.001). Similarly the number of PET scans undertaken increased from 85 to 115 (*P* = 0.005). In a minority of patients ERCP or Octreotide scans were performed where indicated.

The total number of investigations performed increased significantly during the study period from 442 in the first half to 525 in the second. Similarly, the median number of scans performed per patient increased from two (1–4) to three (1–4) (*P* < 0.001).

### 3.3. Correlation of MDT Assessment with Operative Findings

A decision not to resect was made in 21 patients (4.8%) either because of peritoneal disease, tumour progression or because no malignant lesion could be identified (Table 2).

There was no change in the rate of nonresection over time (10/219 versus 11/219). MDT assessment of operability was most accurate for CRM where only 7/270 patients (2.6%) were not resected and least accurate for patients with hilar cholangiocarcinomas where 4/23 patients were not resected (*P* < 0.001).

### 3.4. Correlation of MDT Diagnosis with Final Pathology

Of the 438 patients operated on in this period 42 individuals were identified whose final histological diagnosis was different to the outcome of the MDT discussion (9.6%) (Table 1). There was no change in the rate of discrepant diagnosis over time (23/219 versus 19/219) (Table 3). The median number of lesions per patient was one in both the first (range 0–9) and second (range 0–20) halves of the series (*P* = 0.057). Similarly there was no difference in maximum tumour size with a median of 35 mm (range 6–210) in the first half and 35 mm (range 3–230) in the second (*P* = 0.936). The median number of imaging modalities used was three in patients with discrepant diagnoses compared to two in those with correct diagnoses (*P* = 0.003). The only difference occurred in the use of MRI where 31/42 (73.8%) patients with discrepant diagnoses had additional MRI compared to 196/396 (49.5%) patients where the diagnosis was correct (*P* = 0.003). In total twenty-two patients (5%) underwent hepatic resection for what proved to be benign disease having been diagnosed with malignancy preoperatively. The difficult areas of MDT assessment fell into the following categories.

### 3.5. Hepatocellular Cancer

Thirteen of 44 patients diagnosed as having hepatoma at MDT and proceeding to resection had different histological diagnoses after surgery, of which three were benign. There was no significant difference in the rate of discrepant diagnosis in those with and without a history of chronic liver disease (CLD) (6/19 versus 7/25) (Table 4). In six patients with CLD the final histology revealed a mixed type of tumour with features of both hepatoma and cholangiocarcinoma. For the purposes of this study these have been classed as correct diagnoses.

### 3.6. Cholangiocarcinoma of Major Hepatic Duct

All patients with suspected cholangiocarcinoma of a major hepatic duct underwent cholangiography (percutaneous, endoscopic, or MR) in addition to cross-sectional imaging. Seven of 28 patients diagnosed with cholangiocarcinoma at MDT had a different histological diagnosis after resection (Table 3). There was no significant difference in the rate of incorrect diagnosis in those who presented with obstructive jaundice (3/19) and those without (4/9). Of those patients diagnosed with cholangiocarcinoma without obstructive jaundice, the diagnosis was confirmed in five patients on final histology.

### 3.7. Colorectal Metastases

All patients diagnosed with CRM had a history of colorectal cancer, but 10 (3.6%) had different histological diagnoses after resection (Table 3), of which six were benign. Six of these were metachronous lesions and four were synchronous with their colorectal cancer diagnosis (*P* = 0.539).

### 3.8. Solid Liver Lesions with No History of Chronic Liver Disease or Primary Malignancy

Thirty-four patients underwent resection of peripheral liver lesions (including hepatomas) with no history of CLD or primary malignancy of whom 13 had discrepant diagnoses (Table 4). 

Peripheral cholangiocarcinoma was rarely diagnosed correctly preoperatively. Of eleven patients with a diagnosis of peripheral cholangiocarcinoma at histology, only two had been diagnosed correctly preoperatively, both by percutaneous biopsy. The remainder were inaccurately diagnosed as hepatomas or metastases (Table 3).

### 3.9. Adenoma/FNH/Hepatocellular Carcinoma

A group of 10, predominantly young, female patients (median age 33, range 33–63) was identified in whom the MDT differential list included FNH, adenoma, or hepatocellular carcinoma. After resection all patients had a histological diagnosis that was included in the alternatives made at MDT. In five patients histology revealed hepatic adenoma, four revealed FNH, and one a hepatoma.

## 4. Discussion

This study reveals a number of important features of the MDT assessment of patients with focal liver lesions during the six-year development of a regional HPB unit. Firstly there has been a 50% increase in the number of imaging modalities used in the assessment of these patients over a short time interval. This has been caused by an increased utilisation of PET scans and MRI due to an increased awareness of their role and improved access. Although PET scans have poor sensitivity for detecting multiple liver lesions, they are valuable in the preoperative assessment of patients with hepatic CRM to exclude extrahepatic disease [10, 11]. MRI scans with diffusion-weighted imaging have been shown to have greater sensitivity than CT in the detection of CRM [8, 12], hepatoma  [13], and metastatic NET [14], although these scans have only been available to this department since 2011. The policy of this unit is not to biopsy potentially resectable liver lesions due to the potential risk of tumour seeding [15, 16].

In this series 21 patients (5%) did not undergo surgical resection, and the rate of non-resection did not change significantly over time. The rate of non-resection of liver lesions following assessment has been described previously with reported rates of 3–12% [17, 18]. The commonest cause of non-resection in our series was disease progression. The time interval between imaging and surgery may have a major impact on this outcome, limiting the value of modern imaging. Peritoneal disease was noted in seven of the unresected patients, which is not readily identified by any imaging modality [19].

The highest rate of discrepancies in our series occurred in the group of patients with focal liver lesions without a history of chronic liver disease or primary cancer. This finding emphasises the importance of assessing imaging in the context of the clinical history (13/34). Two observations arise from this group of significance in clinical practice. Firstly the majority of patients (5/6) diagnosed with metastases of unknown origin (MUO) have defined histology after resection, of which the most common is peripheral cholangiocarcinoma. These lesions typically have hypovascular appearances on imaging with ring-like enhancement [20] and can easily be misdiagnosed as colorectal or breast metastases [21]. Recently published guidelines for the management of MUO recommend a range of chemotherapy regimens [22], none of which have been shown to be of benefit in the treatment of cholangiocarcinoma, whereas surgical resection of peripheral cholangiocarcinoma is of proven benefit [23] but is rarely appropriate in the treatment of MUO. Similarly 4/25 patients diagnosed as having hepatoma in this setting are ultimately shown to have peripheral cholangiocarcinoma. Peripheral cholangiocarcinoma is less common than hepatocellular carcinoma [24] which may lead to a low index of suspicion in MDT diagnosis.

In patients with a history of CLD and focal liver lesions, there remains a high rate of patients found not to have hepatoma after excision (7/19). These include neuroendocrine metastases which are hypervascular lesions having similar radiological appearances to hepatoma. This has implications for this patient group where treatment is often recommended without a histological diagnosis. 

The commonest indication for liver resection in our series has been CRM, and the rate of discrepant diagnoses for this group is low (3.6%). The most common alternative diagnosis after resection in this group was haemangioma. The radiological characteristics of this group have been described elsewhere [25] and can be difficult to distinguish from metastases. Interestingly two patients in this group were found to have breast cancer metastases after primary breast surgery two and ten years previously. Breast metastases can have similar radiological features to CRM and can occur many years after the primary diagnosis. A further breast metastasis occurred as an obstructing lesion of the left hepatic duct sixteen years after primary surgery and was diagnosed as a hilar cholangiocarcinoma. 

The high rate of discrepant diagnoses in patients with major duct cholangiocarcinoma has been shown previously [26–28]. These lesions are usually sclerosing adenocarcinomas causing biliary obstruction and are often not visible as a mass lesion [20]. In this situation the presence of the lesion is inferred by the radiological finding of ductal dilation along with clinical features of obstruction. The most common alternative diagnosis in this series was ductal fibrosis. This condition may be a manifestation of an autoimmune process and can have similar radiological features to cholangiocarcinoma [29]. Peribiliary cysts can often be diagnosed preoperatively by the presence of multiple cysts but can also mimic cholangiocarcinoma [20] as in the two cases experienced in this series. The most difficult lesions to assess and make treatment recommendations for are peripheral ductal lesions which do not cause jaundice but are found coincidentally or cause cholestasis. In these patients often the only finding is a short segment of dilated intrahepatic duct. In this series 5/9 of these patients were found to have a cholangiocarcinoma on final histology, and surgery for these lesions is therefore justified, particularly as these lesions can usually be resected safely without the need for resection of the extrahepatic biliary tree.

A particularly difficult group of patients to assess and make treatment recommendations for is the group of predominantly young women with primary liver lesions where the differential diagnosis includes hepatoma, adenoma, and focal nodular hyperplasia. These lesions are usually single but may be multifocal and often occur on a background of obesity or oral contraceptive use [30]. In this series 6/10 lesions were shown to be neoplastic on final histology (adenoma or hepatoma) and surgery appears justified in this patient group. 

Overall 5% of patients underwent surgery for misdiagnosed benign lesions, which is similar to earlier experience [31]. The most common benign lesions were haemangiomas which can be hypo-, iso-, or hyperattenuating on imaging and can sometimes increase in size [25], making distinction from malignant tumours difficult.

In conclusion approximately 10% of patients proceeding to surgery following discussion at the HPB MDT are subsequently shown to have an inaccurate diagnosis and 5% are understaged. Despite an increase in the number of imaging modalities used, there has been no change in this rate over time. These discrepancies must be considered in the context of the risk of overstaging resectable disease or misdiagnosing malignant lesions as benign. 

## Figures and Tables

**Table 1:** MDT indications for resection and number with discrepant histological diagnoses.

Primary MDT diagnosis	Number (%)		Median age (range)		Male/female	Discrepant diagnosis (%)	
Colorectal liver metastases (CRM)	279	(64)	67	(33–90)	176/103	10	(3.6)
Hepatoma	44	(10)	63	(33–84)	31/13	13	(30)
Hilar cholangiocarcinoma	28	(7)	67	(32–77)	14/14	7	(25)
Other metastases	24	(5)	62	(32–76)	8/16	1	(4)
Gall bladder carcinoma	20	(5)	61	(41–82)	5/15	1	(5)
Neuroendocrine tumour (NET)	11	(3)	51	(41–77)	8/3	0	—
Metastasis of unknown origin	6	(1)	63	(43–73)	4/2	5	(83)
Biliary cystadenoma	6	(1)	34	(21–43)	0/6	0	—
Focal nodular hyperplasia (FNH)	5	(1)	34	(30–38)	0/5	0	—
Hepatocellular adenoma	4	(<1)	31	(30–39)	0/4	0	—
Benign cyst	3	(<1)	52	(47–65)	0/3	1	(33)
Breast metastases	3	(<1)	67	(45–78)	0/3	3	(100)
Peripheral cholangiocarcinoma	3	(<1)	70	—	2/1	1	(33)
Primary sarcoma	1	(<1)	71	—	0/1	0	—
Haemangioma	1	(<1)	33	—	0/1	0	—

Total	438		65	(21–90)	248/190	42	(9.8)

**Table 2:** Reasons for nonresection.

Final diagnosis	Number (%)		Peritoneal disease	Disease progression	No/benign disease
Colorectal metastases (CRM)	7/270	(2.6)	4	3	0
Hepatoma	2/33	(6)	0	2	0
Hilar cholangiocarcinoma	4/23	(17)	0	4	0
Gall bladder carcinoma (GBC)	2/19	(11)	2	0	0
Other metastases	3/30	(10)	1	2	0
Neuroendocrine tumour (NET)	1/13	(8)	0	1	0
Haemangioma	1/9	(11)	0	0	1
Normal liver	1	—	0	0	1

Total	21	(4.8)	7	12	2

**Table 3:** Discrepant diagnoses in 42 patients.

MDT diagnosis	Histological diagnosis																	
	Total discrepant	Angiomyolipoma* (1)	Benign cyst* (4)	Benign fibrosis* (3)	Bile duct papilloma* (1)	Breast metastasis (3)	Peripheral cholangiocarcinoma (11)	CRM (270)	FNH* (6)	Focal fat* (2)	Haemangioma* (9)	Hepatoma (34)	NET (13)	No lesion* (2)	Sarcoma (4)	Chronic inflammation* (1)	Ovarian metastasis (5)	Xanthogranulomatous cholecystitis* (1)
Hepatoma (44)	**13**	1	—	—	—	—	5	1	—	1	2	—	2	—	—	1	—	—
Colorectal metastases (CRM) (279)	**10**	—	—	—	—	2	1	—	—	—	4	1	—	2	—	—	—	—
Hilar cholangiocarcinoma (31)	**7**	—	2	3	1	1	—	—	—	—	—	—	—	—	—	—	—	—
Metastases of unknown origin (6)	**5**	—	—	—	—	—	3	—	—	—	1	—	—	—	1	—	—	—
Breast metastases (3)	**3**	—	—	—	—	—	—	—	—	1	1	1	—	—	—	—	—	—
Peripheral cholangiocarcinoma (3)	**1**	—	—	—	—	—	—	—	1	—	—	—	—	—	—	—	—	—
Anal metastases (7)	**1**	—	—	—	—	—	—	—	—	—	—	1	—	—	—	—	—	—
Benign cyst (3)	**1**	—	—	—	—	—	—	—	—	—	—	—	—	—	—	—	1	—
Gall bladder carcinoma (20)	**1**	—	—	—	—	—	—	—	—	—	—	—	—	—	—	—	—	1

Total	**42**	**1**	**2**	**3**	**1**	**3**	**9**	**1**	**1**	**2**	**8**	**3**	**2**	**2**	**1**	**1**	**1**	**1**

Total number of each diagnosis in the series (438) shown in brackets.

All MDT diagnoses of neuroendocrine tumours (NET) (11), focal nodular hyperplasia (FNH) (5), biliary cystadenoma (6), primary sarcoma (1), and haemangioma (1) were confirmed on histology.

*Benign pathology.

**Table 4:** MDT and histological diagnoses of 34 patients with peripheral liver lesions and no history of CLD or malignancy.

MDT diagnosis	Histology								
	Hepatoma	Peripheral cholangio-carcinoma	Haem-angioma	Neuroendocrine tumour	Metastasis of unknown origin (MUO)	Hepatic sarcoma	Focal nodular hyperplasia	Fat	Total
Hepatoma	18	4	1	1	—	—	—	1	25
Metastases of unknown origin	—	3	1	—	1	1	—	—	6
Peripheral cholangiocarcinoma	—	2	—	—	—	—	1	—	3

Total	18	9	2	1	1	1	1	1	34
